# Critical Roles of ELVOL4 and IL-33 in the Progression of Obesity-Related Cardiomyopathy via Integrated Bioinformatics Analysis

**DOI:** 10.3389/fphys.2020.00542

**Published:** 2020-06-05

**Authors:** Jun Tao, Yajing Wang, Ling Li, Junmeng Zheng, Shi Liang

**Affiliations:** ^1^Department of Cardiovascular Surgery, Sun Yat-sen Memorial Hospital, Sun Yat-sen University, Guangzhou, China; ^2^Department of Otorhinolaryngology, Sun Yat-sen Memorial Hospital, Sun Yat-sen University, Guangzhou, China

**Keywords:** ORCM, WGCNA, yellow co-expression module, ELOVL4, IL-33

## Abstract

The molecular mechanisms underlying obesity-related cardiomyopathy (ORCM) progression involve multiple signaling pathways, and the pharmacological treatment for ORCM is still limited. Thus, it is necessary to explore new targets and develop novel therapies. Microarray analysis for gene expression profiles using different bioinformatics tools has been an effective strategy for identifying novel targets for various diseases. In this study, we aimed to explore the potential genes related to ORCM using the integrated bioinformatics analysis. The GSE18897 (whole blood expression profiling of obese diet-sensitive, obese diet-resistant, and lean human subjects) and GSE47022 (regular weight C57BL/6 and diet-induced obese C57BL/6 mice) were used for bioinformatics analysis. Weighted gene co-expression network analysis (WGCNA) of GSE18897 was employed to investigate gene modules that were strongly correlated with clinical phenotypes. Gene Ontology (GO) functional enrichment analysis and Kyoto Encyclopedia of Genes and Genomes (KEGG) pathway analysis were performed on the co-expression genes. The expression levels of the hub genes were validated in the clinical samples. Yellow co-expression module of WGCNA in GSE18897 was found to be significantly related to the caloric restriction treatment. In addition, GO functional enrichment analysis and KEGG pathway analysis were performed on the co-expression genes in yellow co-expression module, which showed an association with oxygen transport and the porphyrins pathway. Overlap analysis of yellow co-expression module genes from GSE18897 andGSE47022 revealed six upregulated genes, and further experimental validation results showed that elongation of very-long-chain fatty acids protein 4 (ELOVL4), matrix metalloproteinase-8 (MMP-8), and interleukin-33 (IL-33) were upregulated in the peripheral blood from patients with ORCM compared to that in the controls. The bioinformatics analysis revealed that ELOVL4 expression levels are positively correlated with that of IL-33. Collectively, using WGCNA in combination with integrated bioinformatics analysis, the hub genes of ELVOL4 and IL-33 might serve as potential biomarkers for diagnosis and/or therapeutic targets for ORCM. The detailed roles of ELVOL4 and IL-33 in the pathophysiology of ORCM still require further investigation.

## Introduction

The number of overweight and obese individuals has dramatically increased. In China, more than 10% of the adult population is obese (He et al., 2017; Qasim et al., 2018; Wang and Ren, 2018). While in America, the prevalence of obesity among adults is about 39.6% (Hales et al., 2018). China is the most populous country with a total population of 1.4 billion compared to 0.32 billion in America (in 2018). Although the prevalence of obesity is much higher in America than in China, the obese population is comparable in these two countries. In 2014, China ranked first worldwide, with 43.2 million obese men and 46.4 million obese women (Collaboration, 2016).

According to the WHO classification, individuals with a body mass index (BMI) ≥ 30 kg/m^2^ are defined as obese (WHO, 2000). However, the universal BMI criteria are not suitable among diverse Asian populations, as Asian populations show different associations between BMI, percentage of body fat, and health risks than European populations (Consultation, 2004). According to the classification of the Chinese Ministry of Public Health, Chinese individuals with a BMI ≥ 28 kg/m^2^ are defined as obese. Waist circumference is another important indicator for obesity, which provides both independent and additive information to BMI for predicting morbidity and risk of death. As BMI alone is not sufficient to properly assess or manage the cardiometabolic risk, the combination of BMI and waist circumference can identify the highest-risk phenotype of obesity far better than either measure alone (Ross et al., 2020).

Obesity is the second leading cause of preventable death and has been identified as a risk factor for heart diseases, type 2 diabetes, certain types of cancers, and metabolic syndromes (Qasim et al., 2018). Overweight and obesity adversely affect cardiovascular (CV) function and serve as an independent risk factor for both systolic and diastolic heart dysfunction, resulting in heart failure (HF) (Wang and Ren, 2018). Obese women are at higher risk of CV disease (CVD), as increased aldosterone and mineralocorticoid receptor activation, aberrant estrogenic signaling, and elevated levels of androgens are among some of the proposed mechanisms explaining the heightened CVD risk. Except for the traditional CV risk factors, excess weight gain during pregnancy, preeclampsia, gestational diabetes, and menopause are central to designing personalized interventions aimed to curb the epidemic of CVD (Manrique-Acevedo et al., 2020). Maternal obesity (MO) during pregnancy exhibits intergenerational effects by programming offspring to CVD. The animal experiment indicates that MO impairs fetal cardiomyocyte contractility through altered intracellular Ca^2+^ handling, overloading fetal cardiomyocyte intracellular Ca^2+^, and aberrant myofilament protein composition (Wang Q. et al., 2019).

In obesity-related cardiomyopathy (ORCM), obesity affects the cardiac function and remodeling in the aspects of myocardial fibrosis, hemodynamic load, and impaired ventricular contractility, which eventually results in HF (Alpert et al., 2014; Carbone et al., 2017). Leptin exerts profound functions in the regulation of food intake, energy expenditure, glucose metabolism, reproduction, and immune response. Therapeutic applications of leptin in the management of obesity and metabolic syndrome are also discussed (Zhang and Ren, 2015). Autophagy pathway may play a pivotal role in the development of cardiac anomalies induced by obesity (Xu and Ren, 2013; Zhang and Ren, 2016).

*In vivo* study revealed high-fat intake downregulated leptin receptor and PPARγ, insulin signaling, phosphorylation of AMPK, ACC, upregulated GATA-4, ANP, NFATc3, PPARα, m-TOR/p70s6k signaling in obesity cardiomyopathy, while knocking out ET-1 receptor A (ET_A_) attenuated the exception of AMPK/ACC. *In vitro* study indicated high-fat diet-induced hypertrophic and autophagic responses can be abolished by the ETA receptor antagonist (Ceylan et al., 2018). However, direct and indirect pathophysiologic factors related to obesity interplay in ORCM, the underling mechanism of ORCM had not been full studied. Meanwhile, current pharmacological treatments have limited curative effects on the clinical outcomes of ORCM. It is essential to broaden our understanding for the precise mechanisms behind these therapeutic modalities in the management of cardiometabolic diseases (Ren and Zhang, 2018). In this regard, it is urgent to explore the molecular mechanisms underlying ORCM progression and to develop novel therapeutic strategies to prevent the progression of ORCM.

Recently, the system biology analysis of microarrays has become an efficient tool for deciphering possible meaningful genes and pathophysiological pathways of various diseases. Sonne et al. (2017) performed microarray analysis to examine the gene expression profiles in adipocytes from diet-induced obese mice and obese ob/ob mice, and identified nine genes in epididymal adipocytes that are possibly involved in immune type1/type2 balance. Rendo-Urteaga et al. (2015) analyzed 28,869 genes using microarray analysis in peripheral blood mononuclear cells (PBMCs) of obese boys and suggested that changes in the gene expression profile of PBMCs in obese boys may help to understand the weight-loss response. Keustermans et al. (2017) used an Illumina microarray platform to analyze gene expression profiles in sorted monocytes from obese and lean children and revealed that monocyte gene expression in childhood obesity might be correlated with obesity and complexity of atherosclerosis in adults. In terms of cardiomyopathy, Zhao et al. (2018) analyzed the gene expression profiles (GSE3585 and GSE42955) of patients with cardiomyopathy and healthy controls and revealed that 89 differentially expressed genes (DEGs) might be associated with cardiomyopathy. In addition, Li et al. (2018) used four data profiles (GSE5406, GSE26887, GSE42955, and GSE57338) for microarray analysis and revealed a common differential gene expression signature in ischemic cardiomyopathy. However, the systematic and thorough microarray analysis for ORCM is still lacking.

In this study, we constructed a co-expression correlation network using the expression data from GSE18897 (whole blood expression profiling of obese diet-sensitive, obese diet-resistant, and lean human subjects) and identified the DEGs from murine datasets (GSE47022, regular weight C57BL/6, and diet-induced obese C57BL/6 mice) using linear models for microarray (LIMMA) data. Selected co-expression modules and DEGs were further subjected to enrichment analysis of Gene Ontology (GO) and Kyoto Encyclopedia of Genes and Genome (KEGG) pathway. Furthermore, the hub genes derived from bioinformatics analysis were validated in the clinical samples from ORCM patients and lean controls. The present study provides preliminary insights into the molecular mechanisms underlying the progression of ORCM.

## Materials and Methods

### Data Collection and Processing

The dataset (GSE18897) including 80-whole-genome expression profiles of the whole blood of obese diet-sensitive, obese diet-resistant, and lean human subjects; and the dataset (GSE47022) including lean individuals and murine data, including the cardiac samples of parallel regular weight C57BL/6, and diet-induced obese C57BL/6 mice, were downloaded from the GEO database. The GSE18897 series were performed on the GPL570 [HG U133_Plus_2] Affymetrix Human Genome U133 Plus 2.0 Array platform (Affymetrix, Santa Clara, CA, United States); the GSE47022 series were performed on GPL8321 [Mouse430A_2] Affymetrix Mouse Genome 430A 2.0 Array (Affymetrix). The raw data were extracted from the CEL files using the oligo package in the Bioconductor^1^ and subsequently processed using the robust multiarray average algorithm (Carvalho and Irizarry, 2010). The human and mouse genes were matched by Gene database^2^. The gene symbols of probes were annotated using the annotation profiles provided by Affymetrix. The DEGs were detected using the R package LIMMA (Ritchie et al., 2015). The cut-off criteria were set as log2-fold change (log2 FC) ≥ 0.5 and *P* < 0.05.

### Construction of Co-expression Network

Global gene expression profiles and co-expressed genes were identified using weighted gene co-expression network analysis (WGCNA; v1.49) package downloaded from Bioconductor. The possibility of two transcripts to construct a weighted network was determined using the soft-threshold method of the Pearson correlation analysis. Average linkage hierarchical clustering was performed to group transcripts based on the topological overlap dissimilarities in the network connection strengths. The restricted minimum gene number was set to 30 for each module, and a threshold of 0.25 was used to merge the similar modules.

### Enrichment Analysis Using GO and KEGG Pathway Analysis

Functional enrichment analysis of the selected genes was performed using the cluster Profiler package (Ritchie et al., 2015). *P* < 0.05 was considered statistically significant, and the identified significant analyses were sorted by gene counts.

### Protein–Protein Interaction (PPI) Network Construction

The protein–protein interaction (PPI) network of the selected genes was constructed using the search tool for the retrieval of interacting genes (STRING) database, and the threshold of medium confidence ≥ 0.4 was applied in the analysis. The constructed PPI network was visualized using Cytoscape software (v3.6.1^3^).

### Blood Sample Collection

A total of six ORCM patients were recruited between July 2018 and June 2019 in the Sun Yat-sen Memorial Hospital of Sun Yat-sen University. The diagnosis of ORCM was based on previous reports (Lavie et al., 2013). The inclusion criteria for ORCM patients were: (1) age > 18 years old; (2) patients with BMI ≥ 28 kg/m^2^ (Chinese classification of obesity) and class II cardiac function according to the NYHA functional classification; and (3) color Doppler ultrasound examination indicating myocardial injury, including diastolic dysfunction, myocardial hypertrophy, or systolic dysfunction (EF < 60%). Healthy volunteers (six) were recruited during routine physical examination. The inclusion criteria for healthy subjects were: (1) age > 18 years; (2) 18.5 < BMI < 23.9; (3) no hyperlipidemia; and (4) color Doppler ultrasound examination indicating no myocardial injury. Exclusion criteria were: (1) pregnancy or breastfeeding; (2) patients with tumors or infection; and (3) patients having heart valve disease. The peripheral blood was collected from both ORCM patients and healthy controls. The serum was extracted from the peripheral blood and was stored at −80°C until further analysis. All the procedures were approved by the Ethics Committee of Sun Yat-sen Memorial Hospital of Sun Yat-sen University (Ethics approval#:SYSEC-KY-KS-2019-019), and each patient submitted written informed consent in accordance with the Declaration of Helsinki. Clinical characteristics of all patients enrolled are summarized in Tables 1, 2.

**TABLE 1:** Clinical characteristics of patients enrolled.

Characteristics	ORCM (*N* = 6)	Control (*n* = 6)
Age (years)	51.8 ± 14.8	51.5 ± 10.5
Men (*N*,%)	5 (83.3%)	3 (50%)
Women (*N*,%)	1 (16.7%)	3 (50%)
CAD (*N*,%)	1 (16.6%)	0 (0%)
Hypertension (*N*,%)	2 (33.3%)	0 (0%)
Diabetes (*N*,%)	0 (0%)	0 (0%)
BMI (kg/m^2^)	30.9 ± 3.5	24.2 ± 3.5
Echocardiography parameters		
LA (mm)	40 ± 4.4	35 ± 4.9
LVDd (mm)	58.1 ± 5.4	49.3 ± 5.0
IVSTd (mm)	10.5 ± 1.5	8.8 ± 1.1
EF (%)	54.8 ± 16.7	70.3 ± 2.8
Diastolic dysfunction (*N*,%)	2 (33.3%)	0 (0%)
NYHA grading		
Grade I (*N*,%)	2 (33.3%)	6 (100%)
Grade II (*N*,%)	4 (66.7%)	0 (0%)
Grade III (*N*,%)	(0%)	0 (0%)
Grade IV (*N*,%)	(0%)	0 (0%)

*Data are present as mean ± SD; CAD, coronary artery disease; BMI, body mass index; EF, ejection fraction; LA, left atrial; LVDd, left ventricular end diastolic diameter; IVSTd, intel-ventricular scptum end-diastolic thickness; EF, ejection fraction.*

**TABLE 2:** Anthropometric information of patient enrolled.

Group	Sex	CAD	Hypertension	DM	Hight (cm)	Weight (kg)	BMI (kg/m^2^)	LA (mm)	LVDd (mm)	IVSTd (mm)	EF (%)	DS
ORCM group	Male	Yes	Yes	No	168	86	30.47	43	55	9	74	No
	Male	No	Yes	No	175	88	28.73	39	55	12	73	Yes
	Male	No	No	Yes	172	89.5	30.25	38	68	10	30	No
	Female	No	Yes	No	144	60	28.94	46	63	13	38	No
	Male	Yes	Yes	No	160	99	38.67	42	54	9	51	No
	Male	No	Yes	No	175	88	28.73	32	54	10	63	Yes
Control group	Female	No	Yes	No	153	60	25.63	38	48	9	73	No
	Female	No	No	No	150	37	16.44	25	39	7	69	No
	Male	No	No	No	158	64	25.64	35	52	8	70	No
	Male	No	No	No	178	79	24.93	35	54	9	67	No
	Male	No	Yes	No	167	72.5	26.00	36	50	10	75	No
	Female	No	No	No	151	61	26.75	41	53	10	68	No

*CAD, coronary artery disease; DM, diabetes mellitus; BMI, body mass index; LA, left atrial; LVDd, left ventricular end diastolic diameter; IVSTd, intel-ventricular scptum end-diastolic thickness; EF, ejection fraction; DS, diastolic dysfunction.*

### Quantitative Real-Time PCR Analysis (qRT-PCR)

The RNA from the serum was extracted using TRIzol reagent (Invitrogen, Carlsbad, CA, United States) according to the manufacturer’s instructions. The mRNA was reverse transcribed into cDNA using the First Strand Synthesis kit (Thermo Fisher Scientific, Waltham, MA, United States). Real-time PCR was performed on an ABI7900 Real-time PCR system (Applied Biosystems, Foster City, CA, United States) using the SYBR Green Master Mix kit (Takara, Dalian, China). GAPDH was used as the control for mRNA expression. The relevant gene expression levels were calculated using the comparative Ct method. The primers for the relevant genes are summarized in Table 3.

**TABLE 3:** Primer sequences for qRT-PCR.

Genes	Forward (5′–3′)	Reverse (5′–3′)
KCTD12	GCTCGGGCTACATCACCATC	GGTCCCGGCTTTCGTTCAG
CD45	ACCACAAGTTTACTAACGCAAGT	TTTGAGGGGGATTCCAGGTAAT
ZNF383	ATGGCTGAGGGATCAGTGATG	GAAACCAGATTGCCGTAGTTCT
C7orf33	CGGTCCAGGTCAATTTAACTTGT	TTTGGTGGGAGCTGATACAGG
MMP8	TGCTCTTACTCCATGTGCAGA	TCCAGGTAGTCCTGAACAGTTT
CYPB	AAGTCACCGTCAAGGTGTATTTT	TGCTGTTTTTGTAGCCAAATCCT
ELOVL4	GAGCCGGGTAGTGTCCTAAAC	CACACGCTTATCTGCGATGG
SNCA	AAGAGGGTGTTCTCTATGTAGGC	GCTCCTCCAACATTTGTCACTT
IL33	GTGACGGTGTTGATGGTAAGAT	AGCTCCACAGAGTGTTCCTTG
GAPDH	AGGTGAAGGTCGGAGTCAAC	CGCTCCTGGAAGATGGTGAT

### Enzyme-Linked Immunosorbent Assay (ELISA) Analysis

The protein levels of elongation of very-long-chain fatty acids protein 4 (ELOVL4), matrix metalloproteinase-8 (MMP8), and interleukin-33 (IL-33) were determined by respective enzyme-linked immunosorbent assay (ELISA) kits (Abcam, Cambridge, MA, United States) according to the manufacturer’s instructions.

### Statistical Analysis

qRT-PCR and ELISA data were analyzed using GraphPad Prism 5 (GraphPad Software, La Jolla, CA, United States). The data were presented as mean ± standard deviation. Significant differences between groups were analyzed using unpaired *t*-test. ^∗^*P* < 0.05 was considered statistically significant.

## Results

### The Workflow of Study Strategies

**FIGURE 1:**
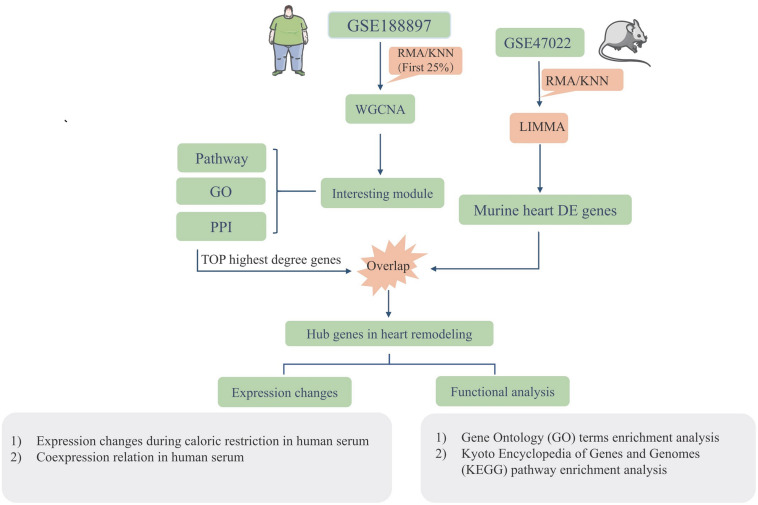
Workflow of the study strategies using datasets including GSE18897 and GSE47022.

Figure 1 illustrates the workflow of the experimental design and data processing. One microarray-based gene expression dataset (GSE18897) included 80-whole-genome expression profiling of the whole blood from obese diet-sensitive, obese diet-resistant, and lean human subjects. The human datasets were used for constructing gene networks with WGCNA, and the expression models were subjected to GO term enrichment and KEGG pathway enrichment analysis. The top highest degree genes were chosen to overlap with the murine heart DEGs. The murine GSE47022 datasets include the cardiac samples of parallel regular weight C57BL/6 and diet induced obese C57BL/6 mice. The GSE47022 datasets were processed using LIMMA to reveal the DEGs in the heart samples. In addition, the overlap hub genes were verified in the clinical samples.

### Construction and Analysis of Gene Co-expression Network With DEGs in Obese Individuals

**FIGURE 2:**
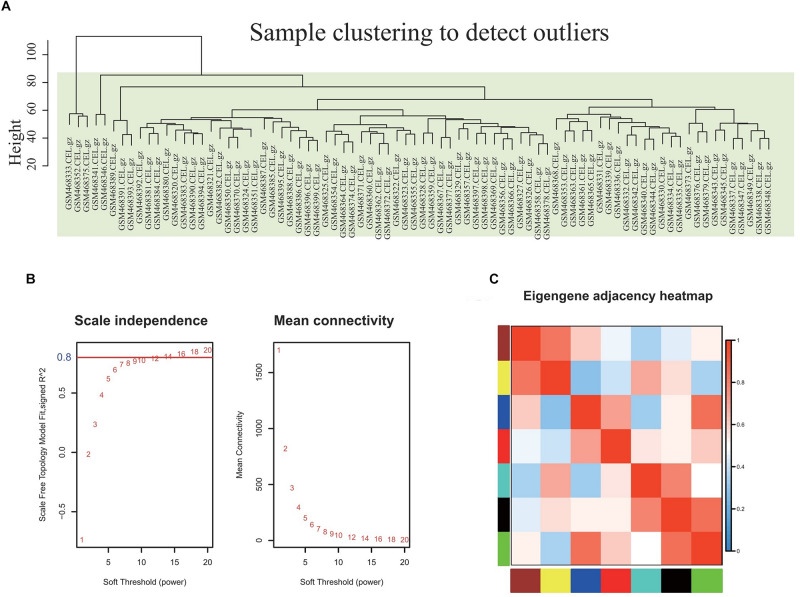
Selection of the proper soft-threshold power β for WGCNA. **(A)** Outliers were determined using sample clustering method, and the analysis was performed according to the expression data of DEGs from whole blood of well-matched obesity and lean individuals. **(B)** The scale-free fit index of network topology was determined by soft-thresholding power analysis. **(C)** Heatmap plot of the adjacencies in the hub gene network. The trait weight was included. Each column and row correspond to one co-expression module hub gene (labeled by color) or weight. In the heatmap, red represents high adjacency (positive correlation) and blue represents low adjacency (negative correlation). Red squares along the diagonal are the meta-modules.

The co-expression network of 5121 genes was analyzed using WGCNA to explore the gene expression network in obesity. First, as shown in Figure 2A, sample clustering was undertaken to detect outliers. The sample clusters were summarized based on the flash-cluster method by combining a heuristic cut-off, and the top 25% most variably expressed genes were selected for analysis (Figure 2A). The power value is a critical parameter and affects the average connectivity degree and independence of the co-expression modules. In this regard, we screened the network topology using different soft thresholding powers, and β = 16 was chosen for further analysis (Figure 2B). Provided that the appropriate soft power parameter was chosen, the eigengenes were used as representative profiles to quantify module similarity by the eigengene correlation (Figure 2C).

**FIGURE 3:**
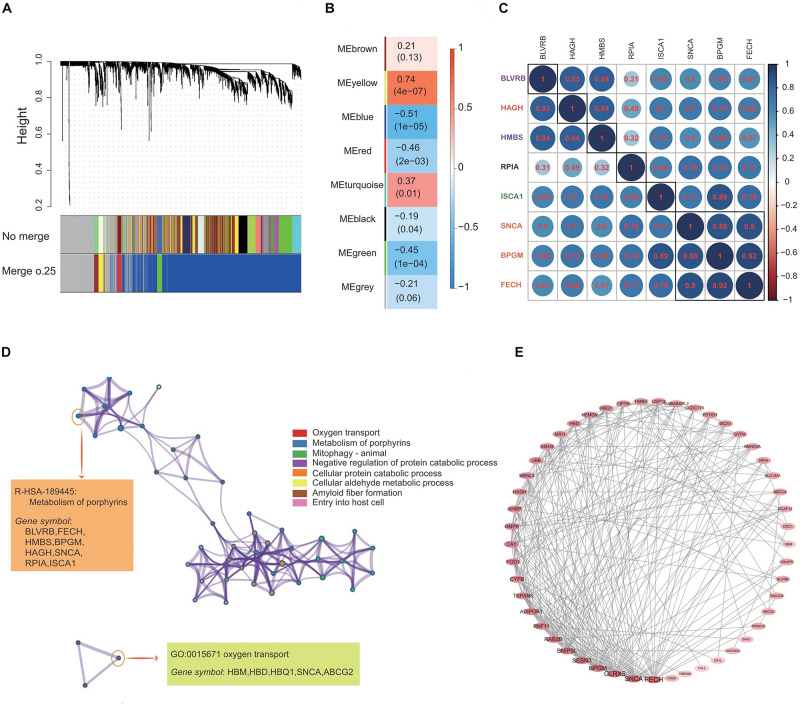
Construction of the co-expression network and identification of the most related co-expression modules. **(A)** Clustering of genes together with assigned module colors. The dissimilarity was based on the topological overlap. The *y*-axis is the distance determined by the extent of the topological overlap. **(B)** Heatmap with each cell containing the *p*-value correlation from linear mixed effects model. Row corresponds to different co-expression modules; column corresponds to traits of caloric restriction treatment. **(C)** The gene correlation heatmap of the genes in yellow co-expression module. Each color circle represents the distance between different genes. **(D)** GO enrichment and KEGG pathway analysis of 633 genes in the yellow co-expression module. **(E)** PPI network construction of the top 50 genes in the yellow co-expression module.

Figure 3A demonstrates the construction of co-expression modules with no merge and merge cut height at 0.25, and the WGCNA analysis, which produced a hierarchical clustering tree (dendrogram) of 5121 genes. A total of eight co-expression modules were generated based on the analysis (Figure 3B). The smallest co-expression module contained 50 genes, while the largest co-expression module contained 3000 genes, and on average, each co-expression module contained 1250 genes. We further determined if any co-expression module was associated with the caloric restriction treatment and investigated the relevance between each module and traits of the caloric restriction treatment. Among eight modules, the co-expression module significance of the yellow co-expression module was higher than that of any other, suggesting it had a greater correlation with the caloric restriction treatment (Figure 3B). The yellow co-expression module showed a positive correlation with the caloric restriction treatment (*r* = 0.74, *p* = 4e-7) and was chosen for further investigation. GO function and KEGG pathway enrichment analyses were performed using DAVID functional annotation to determine the function of these genes in the yellow module. For GO biological processes, genes in the co-expression module were significantly enriched in oxygen transport (Figure 3D); for the KEGG analysis, the genes were mainly enriched in the metabolism of porphyrins (Figure 3D) and the datas can be found in Supplementary Tables S1, S2 in Supplementary Material.

The yellow co-expression module genes were used on the STRING database to clarify high confidence hub genes. The genes were ranked by the PPI nodes, and the top 50 genes were selected (Figure 3E). Among these genes, FECH, SNCA, GLRX5, BPGM, SENSN, BNIP3L, and RAB2B showed the highest connectivity (Figures 3C,E).

### Overlap Analysis of Hub Genes From Human and Mouse DEGs

**FIGURE 4:**
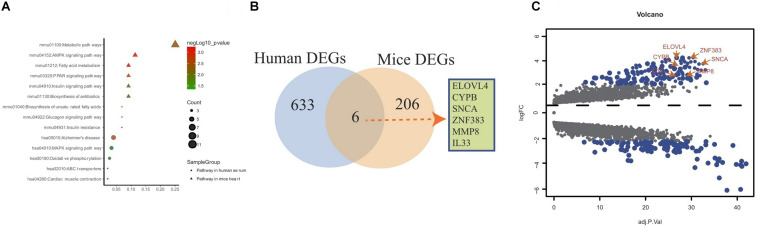
Overlap analysis of hub genes from human and mouse DEGs. **(A)** KEGG pathway analysis of genes from yellow co-expression module and DEGs from mice. **(B)** Venn diagram shows common hub genes between yellow co-expression module and DEGs from mice. **(C)** Volcano plot of yellow co-expression module and DEGs from murine datasets.

The genes in the yellow co-expression module and DEGs from the murine datasets were subjected to KEGG pathway enrichment analysis and the datas can be found in Supplementary Table S3 in Supplementary Material. As shown in Figure 4A, for the murine datasets, metabolic, AMPK, fatty acid, PPAR signaling, insulin signaling, biosynthesis of antibiotics, biosynthesis of unsaturated fatty acids, glucagon signaling, insulin resistance pathways were enriched; for the yellow co-expression module genes, Alzheimer’s disease, MAPK signaling, oxidative phosphorylation, ABC transporters cardiac muscle contraction signaling pathways were enriched. The overlap showed that six common hub genes (ELOVL4, CYPB, SNCA, ZFN383, MMP8, and IL-33) were detected (Figure 4B). The volcano plot results showed that the six common hub genes were all upregulated (Figure 4C).

### Validation of Hub Genes in the Human Clinical Samples

**FIGURE 5:**
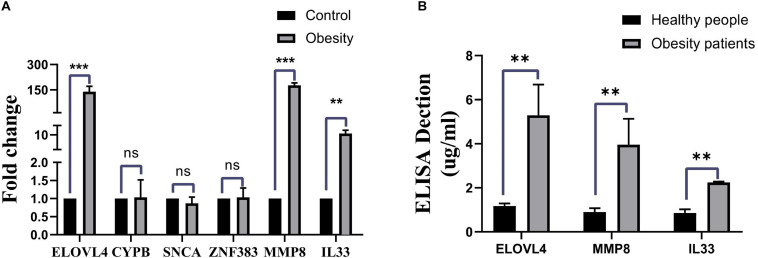
Validation of hub genes in the clinical human samples. **(A)** Gene expression levels of the hub genes (ELOVL4, CYPB, SNCA, ZFN383, MMP8, and IL33) in the human peripheral blood from ORCM patients and healthy controls were determined by qRT-PCR. **(B)** Protein levels of ELOVL4, MMP8, and IL33 in the human peripheral blood from ORCM patients and healthy controls were determined by ELISA assay. *N* = 6; ns = non-significant; **P < 0.01.

To validate the results from bioinformatics analysis, we examined the gene expression levels of these hub genes in human peripheral blood from patients with ORCM and healthy controls. As shown in Figure 5A, the expression levels of ELOVL4, MMP8, and IL-33 were significantly upregulated in the ORCM group compared to normal controls, while no significant difference was detected in the other hub genes. Furthermore, the increased protein levels of ELOVL4, MMP8, and IL-33 in the peripheral blood from patients with ORCM were confirmed using ELISA (Figure 5B).

### Correlation Analysis of ELOVL4 and IL33 Gene Expression Levels Using GEO Database

**FIGURE 6:**
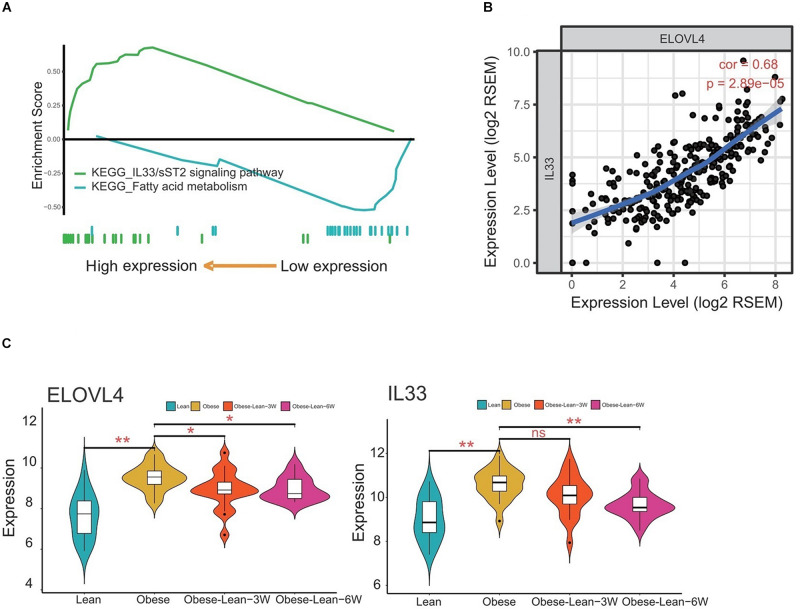
Correlation analysis of ELOVL4 and IL33 gene expression levels. **(A)** KEGG pathway analysis of the hub genes. **(B)** The correlation between ELOVL4 and IL33 expression levels were analyzed using Pearson correlation analysis. **(C)** Analysis of ELOVL4 and IL33 expression levels in lean controls, obese patients, and obese patients received 3- or 6-month period of caloric restriction treatment.

The KEGG pathway analysis revealed that the hub genes derived from the overlap analysis were involved in the fatty acid metabolism and IL33/sST2 signaling pathways (Figure 6A). The correlation analysis showed that ELOVL4 expression levels were positively correlated with IL-33 expression levels (Figure 6B). More importantly, analysis of ELOLV4 and IL-33 using GEO database showed that ELOVL4 and IL33 expression were both upregulated in the obese group when compared to the lean group. Caloric restriction for 3 and 6 months significantly attenuated the increased expression levels of ELOVL4 in the obesity group, while a significant reduction of IL-33 expression levels was observed in the obese patients with a 6-month period of caloric restriction treatment (Figure 6C).

## Discussion

The molecular mechanisms underlying ORCM progression involve multiple signaling pathways and the pharmacological treatment for ORCM is still limited (Khan and Movahed, 2013; Alpert et al., 2014; Tiwari and Ndisang, 2014; Bhatheja et al., 2016). Thus, it is necessary to explore new targets and develop novel therapies. Microarray analysis for gene expression profiles using different bioinformatics tools has been an effective strategy for identifying novel targets for various diseases. In the present study, we performed WGCNA on the series GSE188897 combined with routine bioinformatics analysis and revealed six potential target genes that might be involved in the development of obesity. In addition, the DEGs from GSE47022 were analyzed. The overlap analysis of the hub genes from the WGCNA analysis of GSE188897 and DEGs from GSE47022 revealed six potential genes that may be associated with ORCM. qRT-PCR and ELISA further validated the upregulation of ELOVL4, MMP8, and IL-33 in the patients with ORCM when compared to healthy lean controls. Collectively, in the present study, we used different bioinformatics analytical strategies to explore the potential hub genes related to ORCM development. Further validation studies indicated that ELOVL4, MMP8, and IL-33 might participate in the progression of ORCM.

WGCNA is a newly developed method to identify highly correlated genes in microarrays (Langfelder and Horvath, 2008; Pei et al., 2017). In contrast with the traditional gene-gene correlation co-expression matrices by setting a hard threshold, WGCNA can identify correlated genes using a soft-threshold algorithm and determine the relationship between co-expression modules and phenotypes (Kakati et al., 2019). Thus, WGCNA has been widely used in the study of various diseases, including CVDs (Chen et al., 2016; Wang Y. et al., 2019). Kang et al. (2020) performed the WGCNA in GSE79962 and revealed the potential critical roles of NDUFB5, TIMMDC1, and VDAC3 in the septic cardiomyopathy progression. Using microarray data of coronary artery diseases (CADs; GSE23561), studies detected a co-expression module associated with hypertrophic cardiomyopathy pathway in CAD and found that G6PD and S100A7 were the potential targets (Liu et al., 2016). The GSE18897 datasets were originally generated by Ghosh et al. (2010), and further pathway analysis by gene-set enrichment showed increased transcript levels for genes classified in the oxidative phosphorylation, apoptosis, and ribosome pathways in the obese cohort. In the present study, WGCNA was performed in GSE18897 datasets, and yellow co-expression module was chosen for further GO function and KEGG pathway enrichment analyses. GO function enrichment revealed the genes related to oxygen transport, which was consistent with previous studies showing that transcripts associated with oxygen transport were elevated with the increasing BMI (Rai et al., 2014). Moreover, the genes related to oxygen transport are also upregulated in the peripheral blood from patients with pulmonary hypertension (Cheadle et al., 2012), suggesting the potential involvement of these genes in the ORCM. The KEGG pathway analysis identified enriched genes related to porphyrin metabolism. The porphyrins metabolism is associated with the heme biosynthesis, which is functionally linked to adipogenesis via mitochondrial respiratory activity (Moreno-Navarrete et al., 2017). Our analysis strategies enabled us to identify important genes related to obesity development.

Overlap analysis of hub genes from the yellow co-expression module and DEGs from the murine datasets revealed six upregulated genes. Further experimental validation results showed that ELOVL4, MMP8, and IL-33 were upregulated in the peripheral blood from patients with ORCM compared to the lean controls. The bioinformatics analysis revealed that ELOVL4 expression levels were positively correlated with IL-33 expression levels. These data suggest the potential involvement of ELOVL4 and IL-33 in the pathophysiology of ORCM. ELOVL4 is an elongase that participates in the biosynthesis of very-long-chain (VLC, ≥ C28) saturated fatty acid (VLC-SFA) and polyunsaturated fatty acid (VLC-PUFA) in different tissues (Hopiavuori et al., 2019), and plays a key role in the retinal function (Harkewicz et al., 2012). The increased deposition of long-chain fatty acids in adipocytes is one of the main characteristics of obesity (Walewski et al., 2010). In addition, ELOVL4 is involved in the biosynthesis of VLC fatty acids in the cardiomyocytes (Agbaga et al., 2008). Ge et al. (2012) demonstrated that accumulation of the cardiomyocyte triglyceride and reduction in the ventricular function in obese mice reflected enhanced VLC fatty acid uptake and *de novo* fatty acid synthesis. The involvement of ELOV4 in ORCM might be due to its regulatory effects on the VLC fatty acid synthesis. IL-33 belongs to the IL-1 family of cytokines and acts as an ST2 receptor ligand, and the IL-33/ST2 signaling plays an important role in regulating the progression of cardiomyopathy (Demyanets et al., 2013). Upregulation of IL-33 has been detected in the obese adipose tissues (de Oliveira et al., 2019). Nevertheless, the interaction between ELOVL4 and IL-33 has not been determined, which requires further investigation.

## Limitations

There are several limitations to this study. First, the clinical sample size for validation is relatively small, and future studies are required to increase the sample size and recruit human subjects from multiple clinical centers to confirm the findings. Second, the microarray data were retrieved from diet-induced obese mice, and whether the obese mice had cardiomyopathy is not known, which may lessen the significance of our findings. In terms of the database selection, only one database was chosen for the data analysis, which may have the selection bias, and future studies should consider including more datasets to validate our current findings. Third, whether the caloric restriction treatment in patients with ORCM could restore the expression levels of ELOVL4 and IL-33 requires further determination. Finally, the present study lacks the mechanistic studies regarding the role of ELOVL4 and IL-33 in the ORCM pathophysiology, which may be considered in the future investigation.

## Conclusion

Using WGCNA in combination with integrated bioinformatics analysis, the hub genes of ELVOL4 and IL-33 might serve as potential biomarkers for diagnosis and/or therapeutic targets for ORCM. The detailed roles of ELVOL4 and IL-33 in the pathophysiology of ORCM still require further investigation.

## Data Availability Statement

The datasets generated for this study can be found in the Gene Expression Omnibus (accession: GSE18897, GSE47022).

## Ethics Statement

The studies involving human participants were reviewed and approved by the Ethics Committee of Sun Yat-sen Memorial Hospital of Sun Yat-sen university. The patients/participants provided their written informed consent to participate in this study. Written informed consent was obtained from the individual(s) for the publication of any potentially identifiable images or data included in this article.

## Author Contributions

JZ and SL contributed to study conception and design. JT and YW performed the experiments and analyzed the data. LL interpreted the results. JT prepared figures and drafted the manuscript. All authors edited and revised the manuscript.

## Conflict of Interest

The authors declare that the research was conducted in the absence of any commercial or financial relationships that could be construed as a potential conflict of interest.

## Footnotes

1 http://bioconductor.org/biocLite.R

2 https://www.ncbi.nlm.nih.gov/gene/

3 http://www.cytoscape.org/
