# Supplementary material for: Critical Roles of ELVOL4 and IL-33 in the Progression of Obesity-Related Cardiomyopathy via Integrated Bioinformatics Analysis
Source: Front Physiol. 2020 Jun 5;11:542. doi: 10.3389/fphys.2020.00542 (PMC7291781; doi:10.3389/fphys.2020.00542)
Supplement: Supplementary file 1 [file Data_Sheet_1.PDF]

Table S1. KEGG pathway enrichment analysis

**Term**	**P-Value**	**Genes**
hsa05010:Alzheimer's disease	0.004	NDUFB3, COX7B, SNCA, PPP3R1, COX7C, COX6C
hsa05012:Parkinson's disease	0.012	NDUFB3, COX7B, SNCA, COX7C, COX6C
hsa02010:ABC transporters	0.032	ABCD1, ABCB10, ABCG2
hsa00190:Oxidative phosphorylation	0.052	NDUFB3, COX7B, COX7C, COX6C
hsa03010:Ribosome	0.055	MRPL13, RPS17, RPL34, RSL24D1
hsa04932:Non-alcoholic fatty liver disease (NAFLD)	0.071	NDUFB3, COX7B, COX7C, COX6C
hsa04010:MAPK signaling pathway	0.078	MAP4K3, NRAS, LAMTOR3, PPP3R1, FGF13
hsa04260:Cardiac muscle contraction	0.082	COX7B, COX7C, COX6C
