# Supplementary material for: Critical Roles of ELVOL4 and IL-33 in the Progression of Obesity-Related Cardiomyopathy via Integrated Bioinformatics Analysis
Source: Front Physiol. 2020 Jun 5;11:542. doi: 10.3389/fphys.2020.00542 (PMC7291781; doi:10.3389/fphys.2020.00542)
Supplement: Supplementary file 2 [file Data_Sheet_2.PDF]

Table S2. GO terms enrichment analysis.

a) BP

Term	*P Value*	*Genes*
GO:0006123~mitochondrial electron transport, cytochrome c to oxygen	0.007	COX7B, COX7C, COX6C
GO:0032496~response to lipopolysaccharide	0.020	PPBP, LY96, SNCA, SLPI, CXCL6
GO:0006348~chromatin silencing at telomere	0.037	DPY30, HAT1
GO:0007098~centrosome cycle	0.037	CETN3, TUBE1
GO:0050900~leukocyte migration	0.041	NRAS, CD58, CEACAM8, PROS1
GO:0043312~neutrophil degranulation	0.043	PPBP, ANXA3
GO:0006091~generation of precursor metabolites and energy	0.043	FECH, COX7C, COX6C
GO:1902600~hydrogen ion transmembrane transport	0.056	COX7B, COX7C, COX6C
GO:0006968~cellular defense response	0.057	LGALS3BP, LY96, TRAT1
GO:0042742~defense response to bacterium	0.063	PPBP, CXCL6, IGLL5, ANXA3
GO:0048821~erythrocyte development	0.090	ARID4A, BPGM

b) CC

Term	*P Value*	*Genes*
GO:0005840~ribosome	0.003	MRPL13, MRPL50, RPS17, RPL34, SNCA, RSL24D1
GO:0072562~blood microparticle	0.014	LGALS3BP, SLC4A1, PROS1, IGLL5, HBD
GO:0070062~extracellular exosome	0.017	NDUFB3, MOB1B, SELENBP1, MITD1, RRM2B, DAAM2, LGALS3BP, RPL34, CEACAM8, RNF11, SLC4A1, IGLL5, VASN, RAB2B, MINPP1, CRISP3, LAMTOR3, PDCD10, BPGM, MAN1A1, ANXA3, NRAS, RPS17, CD58, SLPI, VSIG4, PROS1
GO:0005743~mitochondrial inner membrane	0.018	NDUFB3, MRPL13, FECH, MRPL50, COX7B, COX7C, ABCB10, COX6C
GO:0005833~hemoglobin complex	0.071	AHSP, HBD
GO:0042581~specific granule	0.076	CRISP3, ANXA3

c) MF

Term	*P Value*	*Genes*
GO:0004129~cytochrome-c oxidase activity	0.013	COX7B, COX7C, COX6C
GO:0042626~ATPase activity, coupled to transmembrane movement of substances	0.027	ABCD1, ABCB10, ABCG2
GO:0019209~kinase activator activity	0.040	MOB1B, LAMTOR3
GO:0045236~CXCR chemokine receptor binding	0.051	PPBP, CXCL6
GO:0005509~calcium ion binding	0.057	CD69, SNCA, MMP8, PPP3R1, CETN3, MAN1A1, PROS1, ANXA3, RCN2
GO:0042803~protein homodimerization activity	0.062	DPY30, PDCD10, ABCD1, BCL2A1, ABCB10, SLC4A1, MITD1, ACVR1, ABCG2
GO:0016407~acetyltransferase activity	0.073	KAT2B, NAT1
GO:0050431~transforming growth factor beta binding	0.089	VASN, ACVR1
