# Supplementary material for: Critical Roles of ELVOL4 and IL-33 in the Progression of Obesity-Related Cardiomyopathy via Integrated Bioinformatics Analysis
Source: Front Physiol. 2020 Jun 5;11:542. doi: 10.3389/fphys.2020.00542 (PMC7291781; doi:10.3389/fphys.2020.00542)
Supplement: Supplementary file 3 [file Data_Sheet_3.PDF]

Table S3 The KEGG and GO terms enrichment for yellow module genes

Term	P Value	Genes
hsa00860:Porphyrin and chlorophyll metabolism	0.009	FECH, HMBS, BLVRB
GO:0015671~oxygen transport	0.001	HBM, HBQ1, HBD
GO:0055114~oxidation-reduction process	0.007	GLRX5, BLVRB, CREG1, SNCA, ABCC4, GMPR, SESN3
GO:1903507~negative regulation of nucleic acid-templated transcription	0.008	CREG1, SIAH2, MXI1
GO:0042787~protein ubiquitination involved in ubiquitin-dependent protein catabolic process	0.010	RMND5A, RNF11, SIAH2, UBB
GO:0016236~macroautophagy	0.021	GABARAPL2, UBB, OPTN
GO:0043066~negative regulation of apoptotic process	0.043	BNIP3L, SNCA, FHL2, SIAH2, UBB
GO:0044267~cellular protein metabolic process	0.046	SNCA, SIAH2, UBB
GO:0043065~positive regulation of apoptotic process	0.057	BNIP3L, SNCA, UBB, GADD45A
GO:0042493~response to drug	0.059	FECH, SNCA, ABCC4, ABCG2
GO:0006783~heme biosynthetic process	0.060	FECH, HMBS
GO:0000422~mitophagy	0.097	GABARAPL2, CISD2
GO:0005833~hemoglobin complex	0.000	HBM, AHSP, HBQ1, HBD
GO:0005829~cytosol	0.002	CTNNAL1, ODC1, GABARAPL2, HMBS, SNCA, PIP5K1B, BPGM, SNX3, OPTN, GMPR, HAGH, BNIP3L, BLVRB, DNAJC6, YOD1, RPIA, UBB, SIAH2, CA1, HBD
GO:0005737~cytoplasm	0.056	ODC1, GABARAPL2, PLEK2, HMBS, C9ORF78, SNCA, SNX3, OPTN, GMPR, MXI1, SESN3, HAGH, DCAF12, RMND5A, BLVRB, PBX1, MBNL3, UBB, SIAH2, CA1, GADD45A, CR1L
GO:0005759~mitochondrial matrix	0.070	HAGH, ISCA1, GLRX5, FECH
GO:0051537~2 iron, 2 sulfur cluster binding	0.000	ISCA1, GLRX5, CISD2, FECH

GO:0005344~oxygen transporter activity	0.001	HBM, HBQ1, HBD
GO:0008198~ferrous iron binding	0.002	ISCA1, FECH, SNCA
GO:0042626~ATPase activity, coupled to transmembrane movement of substances	0.007	ABCC4, ABCC13, ABCG2
GO:0019825~oxygen binding	0.008	HBM, HBQ1, HBD
GO:0042802~identical protein binding	0.019	GYPA, BNIP3L, SNCA, FHL2, ADIPOR1, RPIA, OPTN
GO:0005515~protein binding	0.029	GYPB, CTNNAL1, C9ORF78, SNCA, PIP5K1B, XK, FHL2, SNX3, MXI1, HEMGN, CISD2, RMND5A, USP12, RNF11, YOD1, RPIA, MBNL3, HBD, RAB2B, GABARAPL2, ODC1, FECH, OPTN, ABCG2, HAGH, BNIP3L, AHSP, HBQ1, PBX1, UBB, SIAH2, CA1, GADD45A
GO:0020037~heme binding	0.058	HBM, HBQ1, HBD
GO:0005506~iron ion binding	0.070	HBM, HBQ1, HBD
GO:0048487~beta-tubulin binding	0.098	GABARAPL2, SNCA
